# Strength, microstructure, and thermal conductivity of the insulation wallboards prepared with rice husk fiber and recycled concrete aggregates

**DOI:** 10.1371/journal.pone.0203527

**Published:** 2018-09-19

**Authors:** Xiaoniu Yu, Linzhu Sun

**Affiliations:** College of Architecture and Civil Engineering, Wenzhou University, Wenzhou, China; Texas A&M Univ, UNITED STATES

## Abstract

This paper intends to evaluate the influence of content of rice husk fiber and cementitious materials on mechanical properties and thermal conductivity of thermal insulation wallboards. Thermal insulation wallboard contained different mass of rice husk fiber was prepared when the weight of cement, fly ash, cellulose ether, naphthalene superplasticizer, and recycled concrete aggregates was equal. Scanning electron microscopy (SEM) shows the internal structure of the insulation wallboards is very dense. Compared to thermal conductivity of blank group (0.9600 W/m·°C), B2 (0.1997 W/m·°C) and C2 (0.1810 W/m·°C) measured by the DRCD-3030 intelligent thermal conductivity tester can meet certain engineering requirements. Average compressive strength, flexural strength, and thermal conductivity of wallboards decreases with content of rice husk fiber increasing when other materials mass are the same. Under the same conditions of curing time and rice husk content, average compressive and flexural strength increase with the increase of the amount of cementitious material.

## 1. Introduction

Concrete materials in human social and economic development are indispensable. With the progress of society and people’s living standards continues to improve. The per capita demand for concrete has increased year by year, indirectly lead to ecological damage, environmental pollution, and other issues become increasingly prominent. Gravel and sand as non-renewable resources have shown a shrink tendency under the huge consumption. To ensure high-quality aggregate supply more difficult, which will inevitably affect the construction and national economic development. With the acceleration of civil engineering construction, huge amounts of construction waste are generated, of which nearly half are waste concrete. The utilization rate of China’s construction waste is less than Europe and the United States. Waste concrete make into recycled aggregates, replacing normal natural aggregates has become an important issue, and researched by scientists around the world [1–23].

The use of recycled concrete aggregate as a building material is now gaining popularity in China’s construction materials. High-performance concrete, stub columns, and beams can be cast by recycled concrete aggregates, and have good mechanical properties according to literature reported [24–27]. However, few studies have been conducted on the production of thermal insulation wallboards through recycled aggregates according to web of science index. In this paper, thermal insulation wallboards were prepared with recycled concrete aggregate and rice husk fiber. And the structure and morphology of the wallboards were characterized by X-ray diffraction (XRD) analysis and scanning electron microscopy (SEM). The mechanical properties of the specimens were tested using pressure/bending test machine. In actual engineering, materials with a thermal conductivity of less than 0.20 W/m·°C are usually called thermal insulation materials [28–30]. The results show B2 and C2 of specimens can meet the actual engineering of strength requirements.

## 2. Experiments and methods

### 2.1. Experimental materials

(1) Cement: The cement used in this experiment was ordinary Portland cement 42.5 (Zhejiang Hongshi Cement CO., LTD, China), as shown in S1 Table. The initial time and final setting time of the cement were 155 and 334 min, respectively. The 3-day compressive strength and flexural strength were 27.8 and 5.2 MPa, respectively. The 28-day compressive strength and flexural strength were 45.5 MPa, and 7.8 MPa, respectively. (2) Fly ash: The fly ash used in this experiment had properties as follows: the thinness (45 μm residue on sieve), water absorption, the loss on ignition, water content, and the sulfur trioxide content were 18.5%, 107%, 5.8%, 0.75%, and 2.6%, respectively. (3) Recycled concrete aggregates: Waste concrete was made into fine aggregate with particle size of less than 3mm through the aggregate crusher in laboratory (Wenzhou construction and demolition waste, China), as shown in S1 Fig. The water absorption of recycled concrete aggregates was 15.19%. (4) Cellulose ether, naphthalene superplasticizer, and rice husk fiber were purchased from Wenzhou Jingheng Chemical Reagents Company, China.

### 2.2. Experimental mix proportion design

The cement, fly ash, concrete powder, cellulose ether, naphthalene superplasticizer, and rice husk fiber were mixed in different proportions to make different kinds of cement-based materials, as shown in Table 1. The consistency of cement-based slurry was adjusted to about 8CM. According to the number of specimens, the actual amount of different types of test materials was determined, as shown in S2 Table. The test dimensions were 70.7mm×70.7mm×70.7mm, 40mm×40mm×160mm, and 30mm×300mm×300mm for 12 groups. Each group of specimens was four and numbered A1 ~ A4, B1 ~ B4, and C1 ~ C4. Number A1 ~ A4, B1 ~ B4, and C1 ~ C4 were one group, respectively.

**Table 1:** Mass of raw materials.

Number	Total gelling material (g)	Cement (g)	Fly ash (g)	Concrete powder (g)	Cellulose ether (g)	Naphthalene superplasticizer (g)	Rice husk fiber (g)
A1	4285.70	3428.56	857.14	10000	3.21	21.43	0.00
A2	4285.70	3428.56	857.14	10000	3.21	21.43	571.67
A3	4285.70	3428.56	857.14	10000	3.21	21.43	786.04
A4	4285.70	3428.56	857.14	10000	3.21	21.43	1000.00
B1	5384.60	4307.68	1076.92	10000	4.04	26.92	0.00
B2	5384.60	4307.68	1076.92	10000	4.04	26.92	615.38
B3	5384.60	4307.68	1076.92	10000	4.04	26.92	846.15
B4	5384.60	4307.68	1076.92	10000	4.04	26.92	1076.97
C1	6666.70	5333.36	1333.34	10000	5.00	33.33	0.00
C2	6666.70	5333.36	1333.34	10000	5.00	33.33	666.67
C3	6666.70	5333.36	1333.34	10000	5.00	33.33	916.67
C4	6666.70	5333.36	1333.34	10000	5.00	33.33	1166.67

### 2.3. Specimens preparation

**Fig 1:**
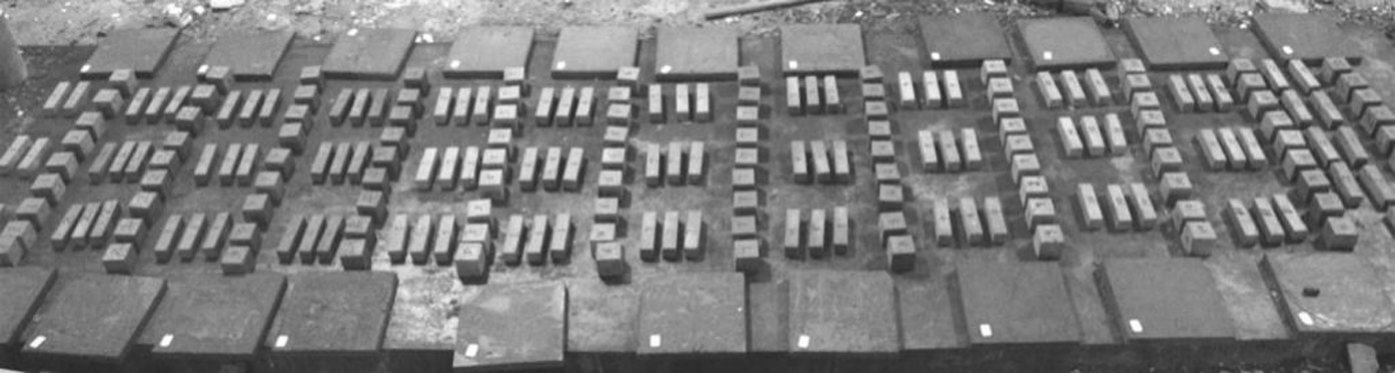
Image of the specimens after demolding.

The contents of the experimental materials were calculated and measured according to the requirements for mix proportion. Recycled concrete aggregates, cement, fly ash, cellulose ether, naphthalene superplasticizer, and rice husk fiber were sequencely added into a small mortar mixer. The mixer was covered and mixed for 2 min to achieve uniform mixing. A certain amount of running water was added to the mixture, and continued stirring for 3 min. The consistency of all cement-based slurries was adjusted to about 8CM. The cement-based slurry was then poured into a particular mold (70.7mm×70.7mm×70.7mm, 40mm×40mm×160mm, and 30mm×300mm×300mm). Specimens were demolded after 3d, as shown in Fig 1. Putting the specimens to a standard curing room at temperature of 20 ± 1 °C and relative humidity of above 95%, and cured for 7, 14, and 28 days.

### 2.3. Characterization

**Fig 2:**
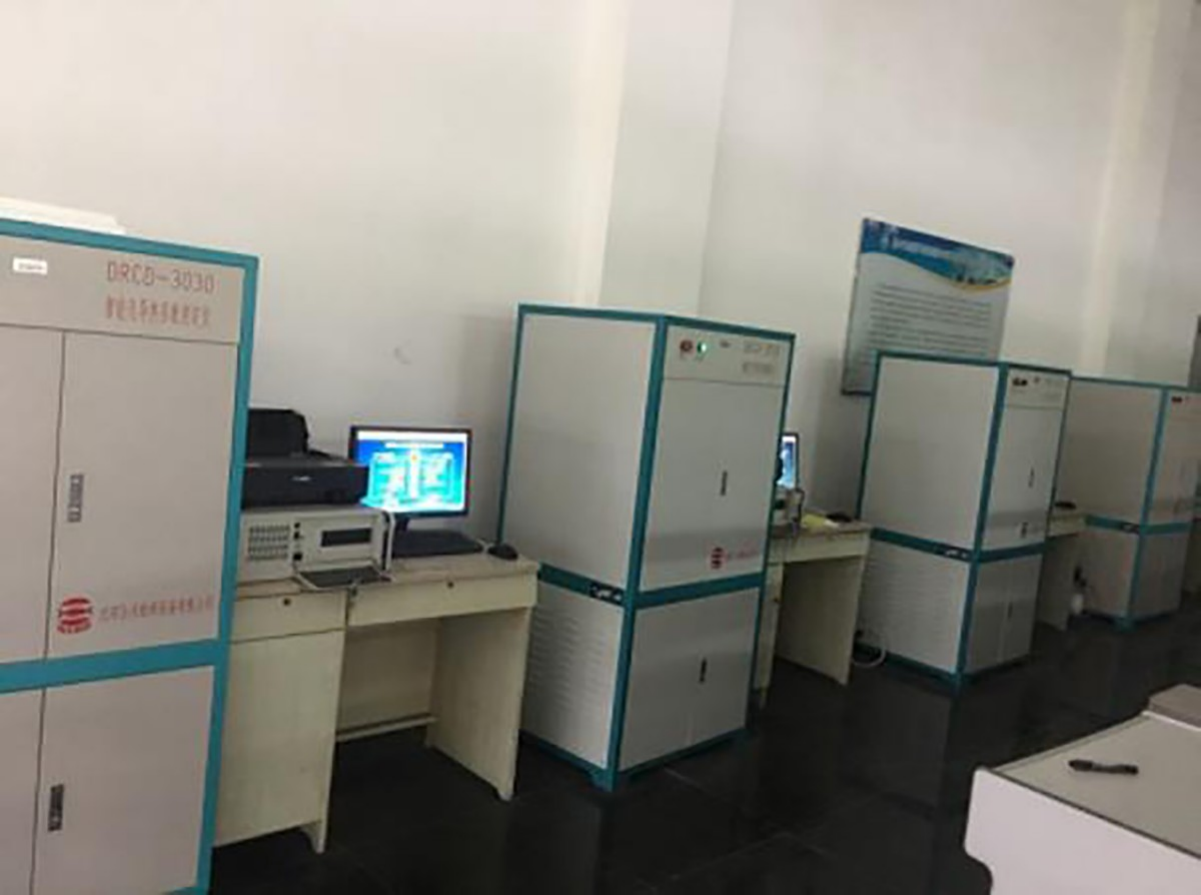
DRCD-3030 intelligent thermal conductivity tester.

The phase composition of products was examined by X-ray diffraction (XRD, Bruker D8-Advance, λ = 1.5406Å, Bruker Company, Karlsruhe, Germany). Scanning electron microscope (SEM, JEOL-7800Prime, operating voltage 5 kV, JEOL, Tokyo, Japan) was used to conduct morphological studies of the samples. The compressive strength and flexural strength of specimens was tested through the ordinary compression/bending test machine. The thermal conductivity of the insulation panels was measured by the DRCD-3030 intelligent thermal conductivity tester (DRCD-3030, Shenyang Hexing Company, Shenyang, China), as shown in Fig 2.

## 3. Results and discussion

### 3.1. XRD pattern and micro-structure of the specimens

**Fig 3:**
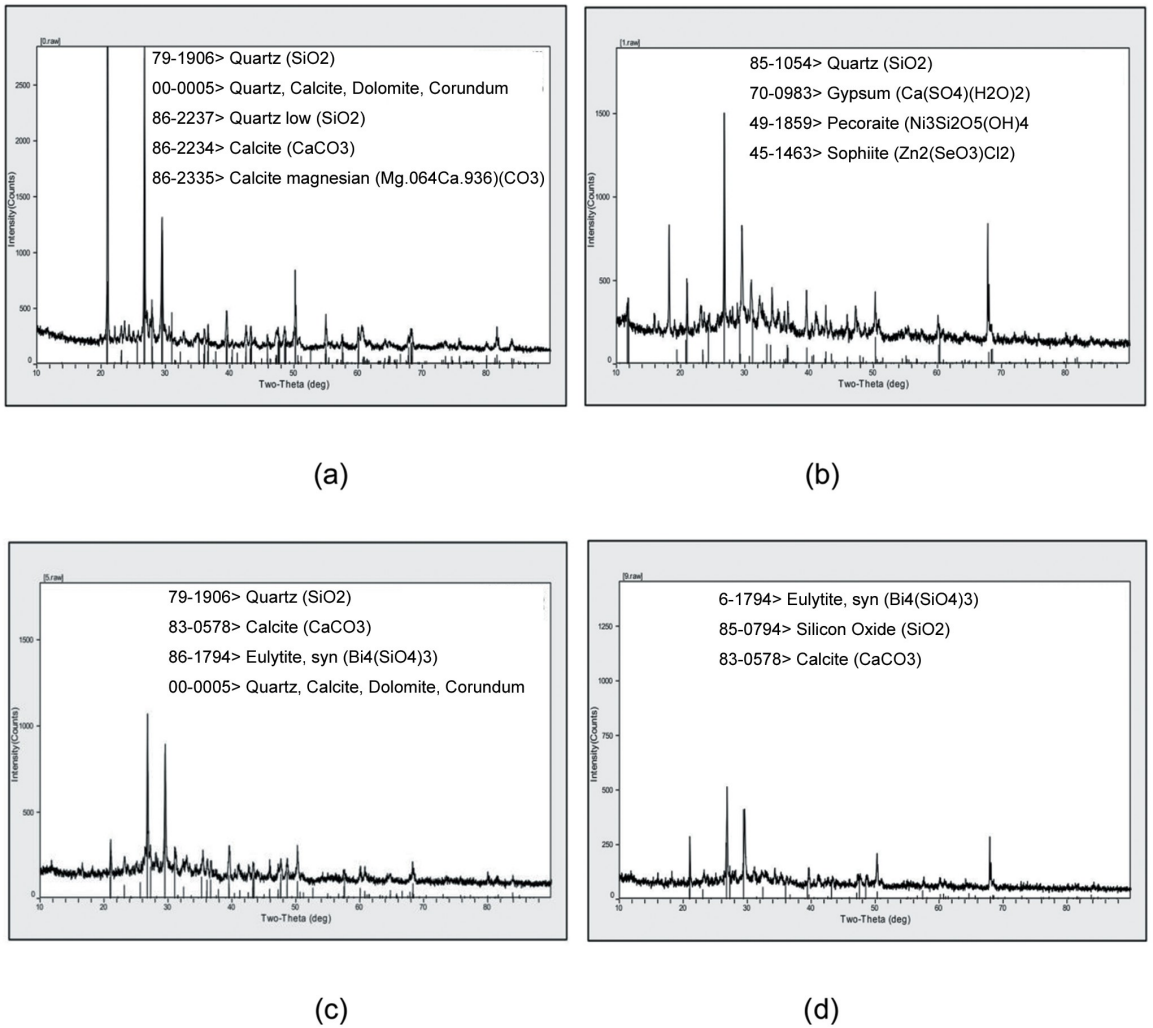
XRD patterns of the specimens: (a) concrete powder, (b) A1 sample, (c) B1 sample, (d) C1 sample.

The composition of concrete powder was analyzed through XRD, as shown in Fig 3. Concrete powder components were mainly a mixture of quartz (JCPDS No. 79–1906), calcite (JCPDS No. 86–2334), Dolomite (JCPDS No. 00–0005), and other impurity materials. The composition of waste concrete is different from the river sand and artificial sand. Amorphous cement hydration products cannot be indexed by the jade 5 program. The constituents of thermal insulation wallboards were analyzed through XRD, as shown in Fig 3a, 3b, 3c, and 3d. The XRD results indicated that A1, B1, and C1 samples were mainly a mixture of quartz (JCPDS No. 79–1906), calcite (JCPDS No. 86–2334), and other mineral admixtures. The peaks of quartz and calcite are from concrete powder. The peaks of cement hydration products are too weak and are submerged by the quartz peaks, so it cannot be indexed by the jade 5 program. Therefore, concrete powder mixed with cement can be made into recycled concrete mortar.

**Fig 4:**
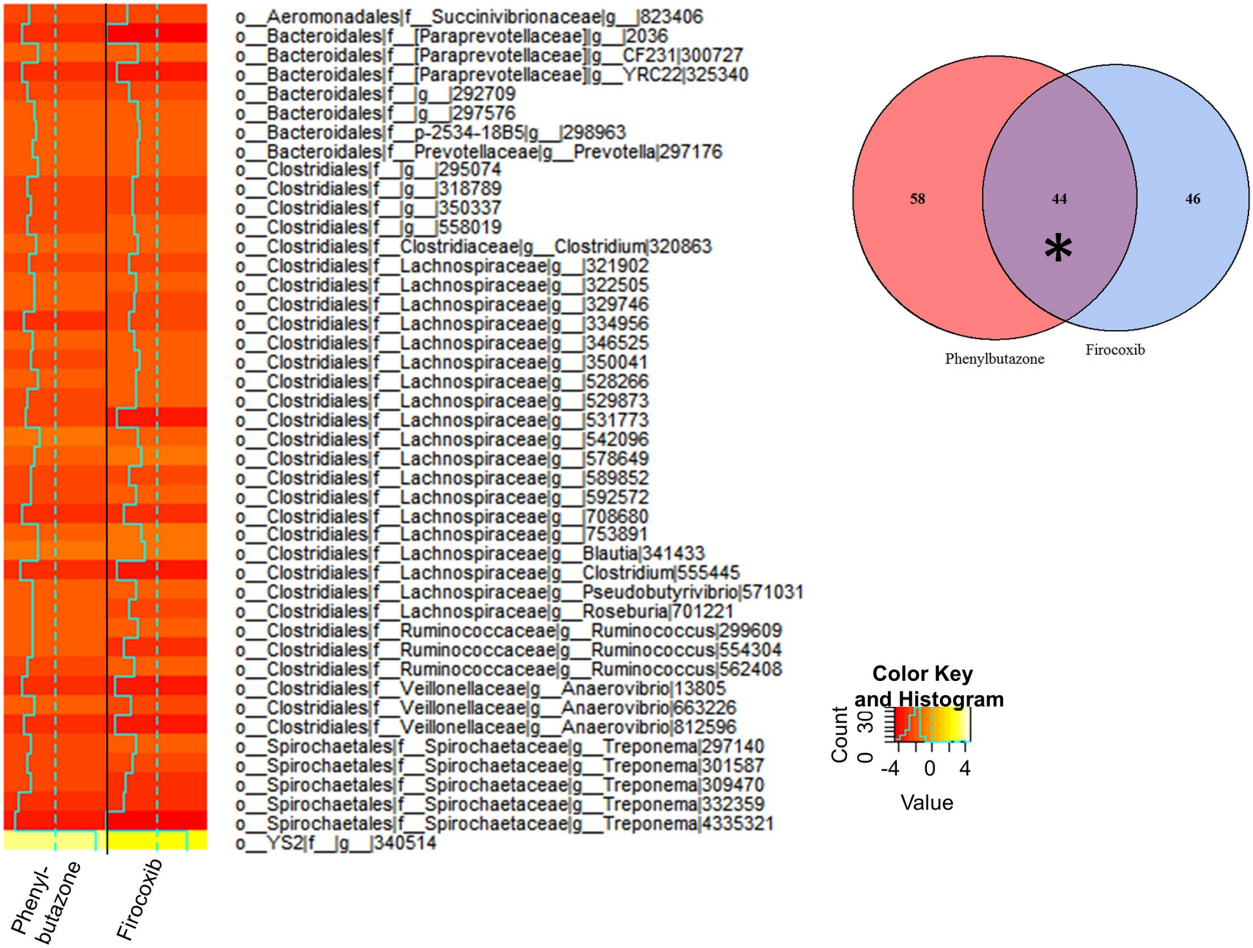
SEM images of the specimens: (a, b) concrete powder, (c, d) A1 sample, (e, f) A2 sample, (g, h) A3 sample, (i, j) A4 sample.

**Fig 5:**
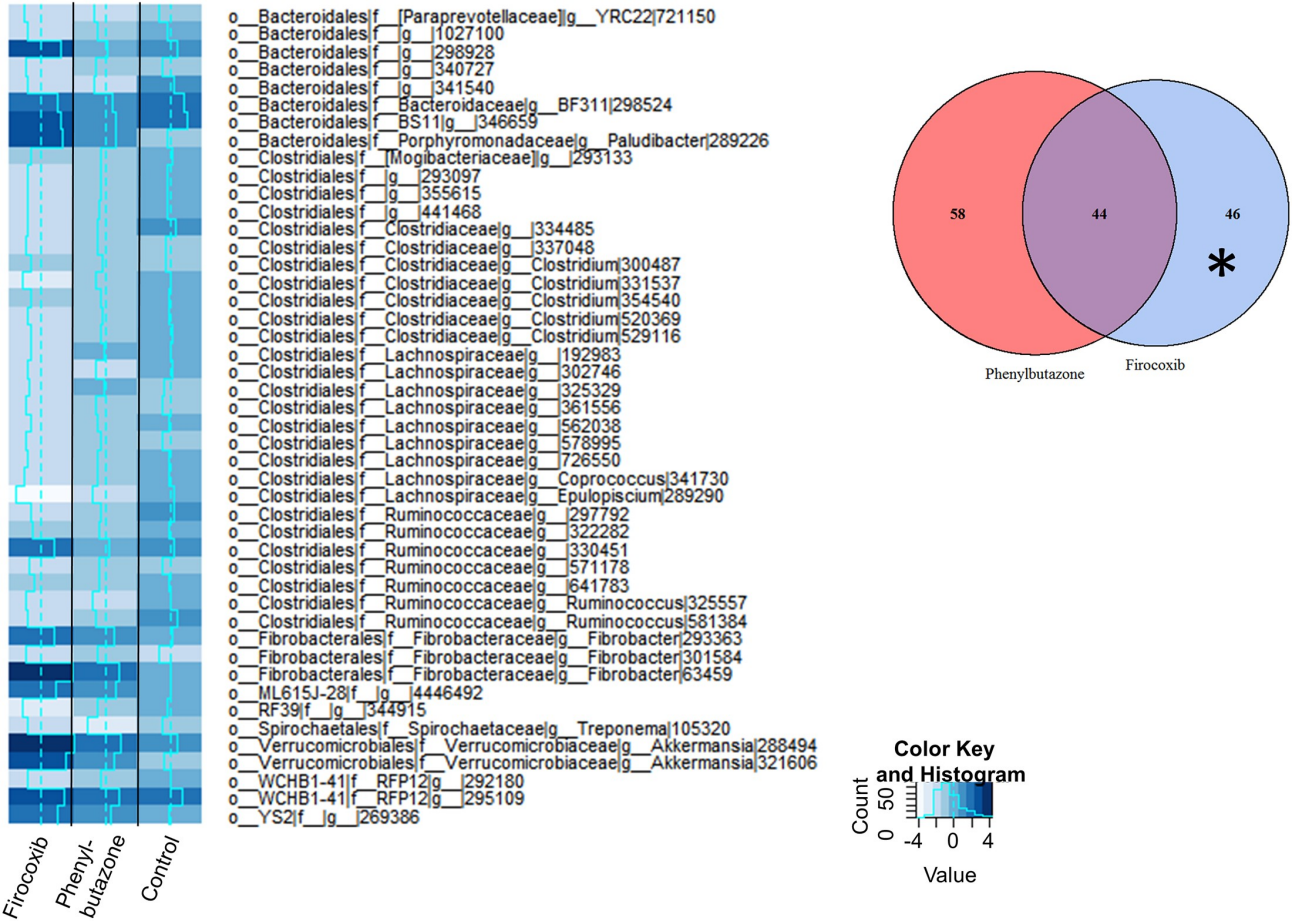
SEM images of the specimens: (a, b) B1 sample, (c, d) B2 sample, (e, f) B3 sample, (g, h) B4 sample.

**Fig 6:**
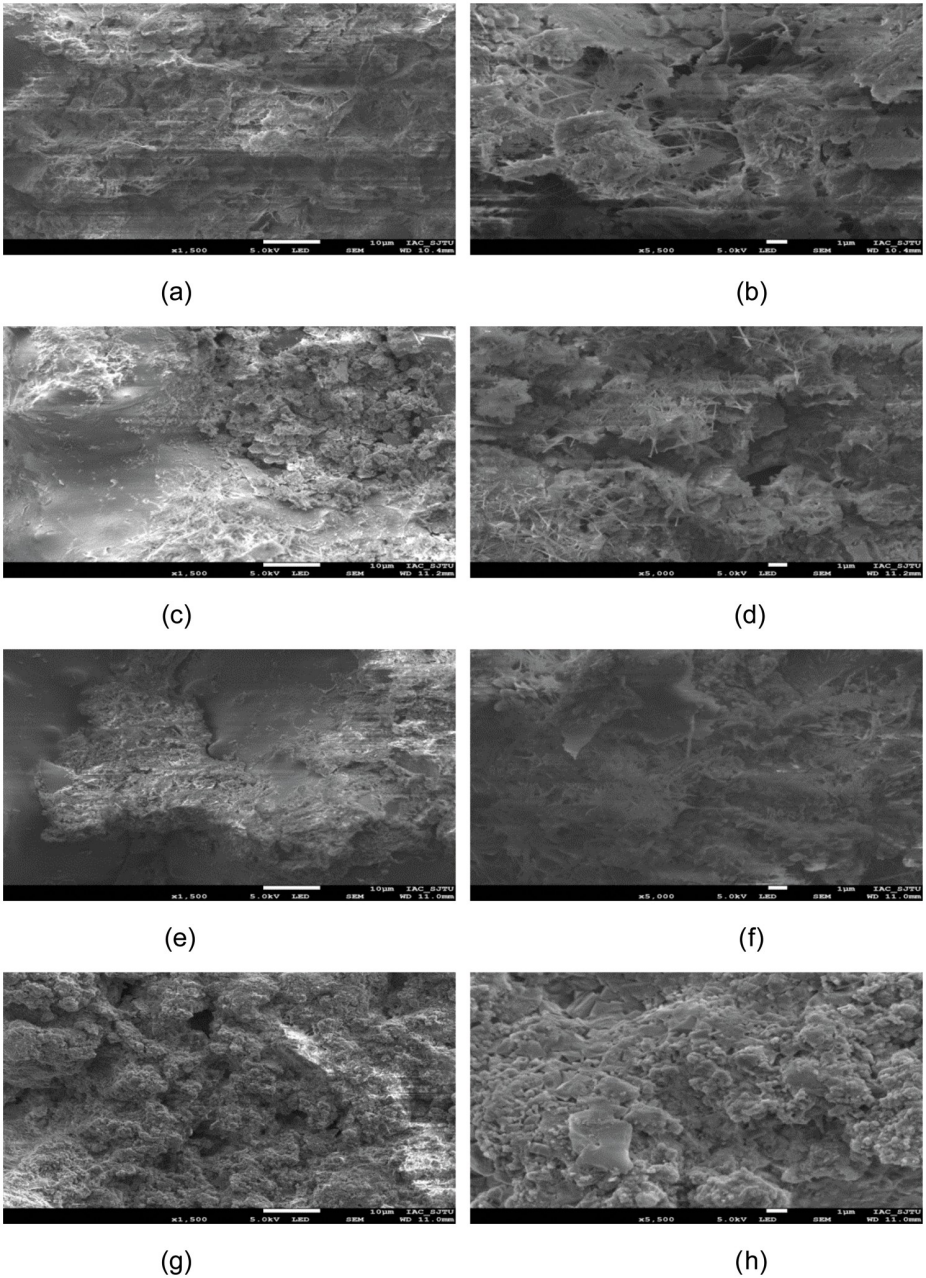
SEM images of the specimens: (a, b) C1 sample, (c, d) C2 sample, (e, f) C3 sample, (g, h) C4 sample.

SEM images of concrete powder are shown in Fig 4a and 4b. SEM images indicate that shape of concrete powder is irregular block structure and rough surface. SEM images of all types of thermal insulation wall boards were shown by Figs 4, 5, and 6. Cement, concrete powder, rice husk, etc. can be well combined together (Figs 4c, 4e, 4g, 4I, 5a, 5c, 5e, 5g, 6a, 6c, 6e, 6g). The morphology for all types of thermal insulation wall boards is similar to each other. Their morphology is mainly needle-like, flaky, spherical, flocculent, and irregular shape. The results also further confirm the morphology of cement hydration products.

### 3.2. Influence of the content of rice husk fiber on compressive strength and flexural strength of the specimens

**Fig 7:**
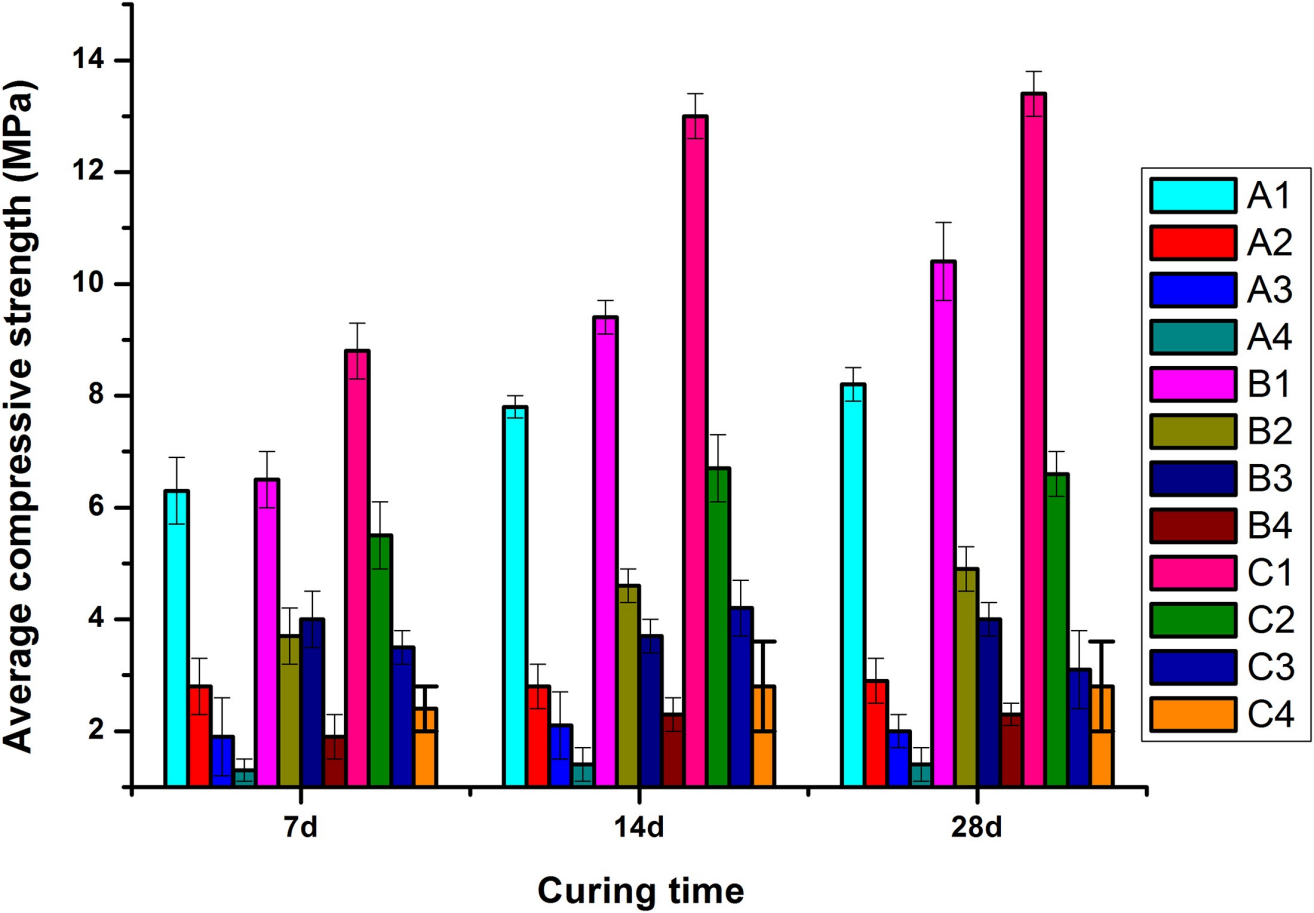
Relationship between curing time and average compressive strength.

**Fig 8:**
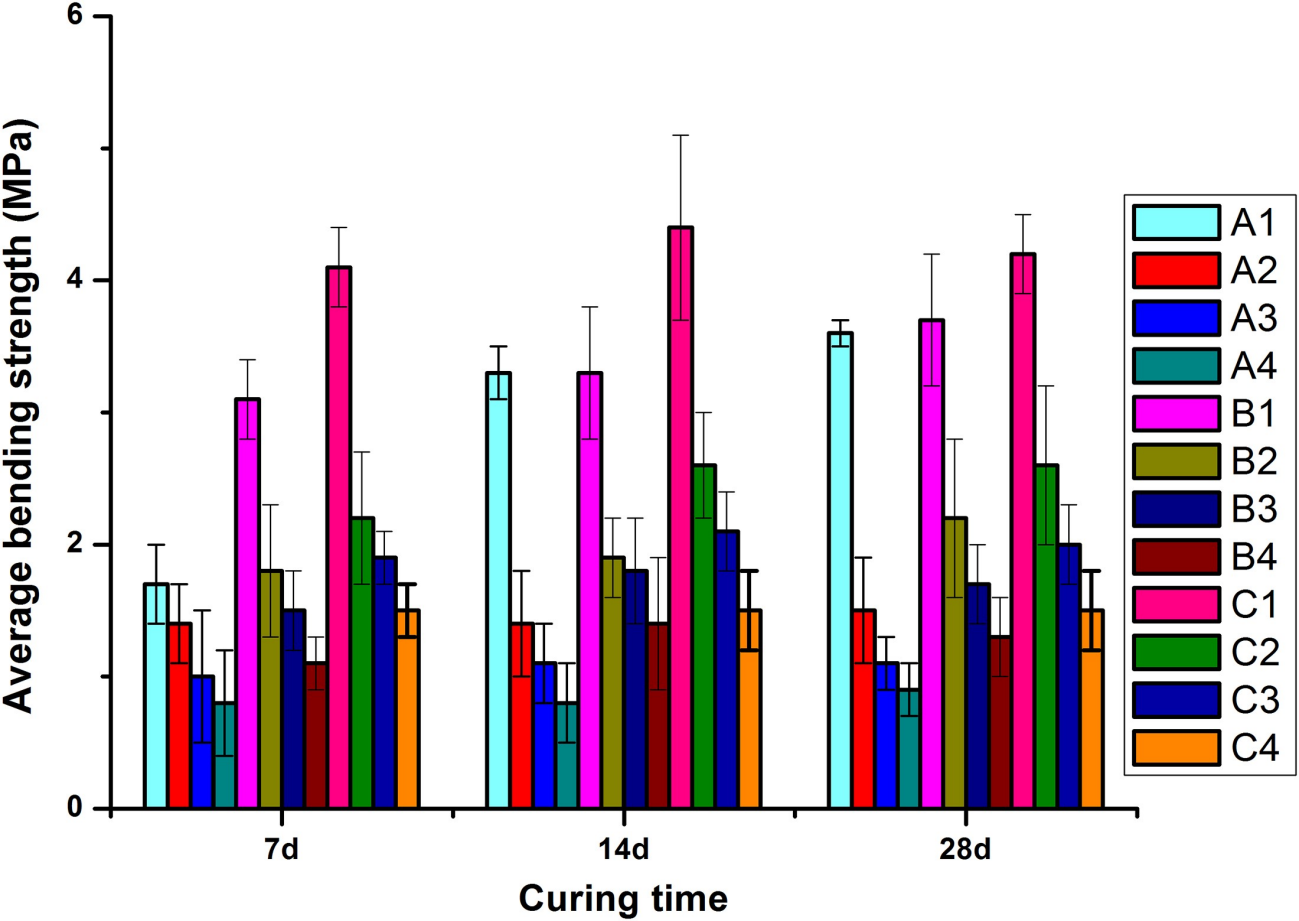
Relationship between curing time and average flexural strength.

Compressive strength and flexural strength of the specimens in the same age decreasing with the increase of the amount for rice husk fiber when the content of cement, fly ash, concrete powder, cellulose ether, and naphthalene superplasticizer are same, as shown Figs 7 and 8. 7, 14, and 28d of average compressive strength for groups A1 ~ A4 were 6.3, 7.8, and 8.2Mpa, 2.8, 2.8, and 2.9Mpa, 1.9, 2.1, and 2.0Mpa, 1.3, 1.4, and 1.4Mpa, respectively (Fig 7). The average compressive strength of A1 and A2 increased with the increase of curing time and the increase of rice hull content. A1 has the largest growth rate. The average compressive strength of A3 and A4 increased with the increase of curing time and the increase of rice husk content, and the increase was 0.1Mpa. The intensity of 14 ~ 28d was basically maintained constant.

7, 14, and 28d of average compressive strength for groups B1 ~ B4 were 6.5, 9.4, and 10.4Mpa, 3.7, 4.6, and 4.9Mpa, 4.0, 3.7, and 4.0Mpa, 1.9, 2.3, and 2.3Mpa, respectively (Fig 7). Average compressive strength of B1 and B2 increased with the increase of curing time. B1 has the largest growth rate. The average compressive strength of B3 decreased firstly and then increased, and the intensities were the same at 7d and 28d, with the increase of curing time. The average compressive strength of B4 was increased by 0.4Mpa at 14 days and the intensity of 14–28 days remained unchanged with the increase of curing time.

For groups C1 ~ C4, average compressive strength at 7, 14, and 28d were 8.0, 13.0, 13.4Mpa, 5.5, 6.7, and 6.6Mpa, 3.5, 4.2, and 3.1Mpa, 2.4, 2.8, and 2.8Mpa, respectively (Fig 7). The average compressive strength of C1 was increased with the increase of curing time, and the growth rate was the largest. With the increase of curing time, the average compressive strength of group C2, C3, and C4 increased corresponding to 7 and 14d, then C2 and C3 were reduced, C4 was maintained.

For the group numbers A1 ~ A4, 7, 14, and 28d of average flexural strength were 1.7, 3.3, and 3.6Mpa, 1.4, 1.4, and 1.5Mpa, 1.0, 1.0, and 1.1Mpa, 0.8, 0.8, and 0.9Mpa, respectively, as shown in Fig 8. The average flexural strength of groups A1-A4 increases with the increasing curing time and content of rice hull. A1 increases the maximum. The average flexural strength of A2 was maintained at 7 ~ 14d with the increase of curing time.

Fig 8 shows 7, 14, and 28d of average flexural strength of groups B1 ~ B4 were 3.1, 3.3, 3.7Mpa, 1.8, 1.9, and 2.2Mpa, 1.5, 1.8, and 1.7Mpa, 1.1, 1.4, and 1.3Mpa, respectively. Average flexural strength of B1 and B2 increased with the increase of curing time. With the increase of curing time and content of rice husk, average flexural strength of groups B3 and B4 increased.

Average flexural strength of groups C1 ~ C4 was 4.1, 4.4, and 4.2Mpa, 2.2, 2.6, and 2.6Mpa, 1.9, 2.1, and 2.0Mpa, 1.5, 1.5, and 1.5Mpa, respectively, as shown in Fig 8. Average flexural strength of C1, C3 increased firstly and then decreased with the increase of curing time, and the increase and decrease was not significant. With the increase of curing time, the average flexural strength of C2 was increased at 7 and 14 d, and the strength of 14 and 28d was kept constant. The average flexural strength of C4 remained unchanged with the increase of curing time. The above changes are related to curing time and rice husk content.

**Fig 9:**
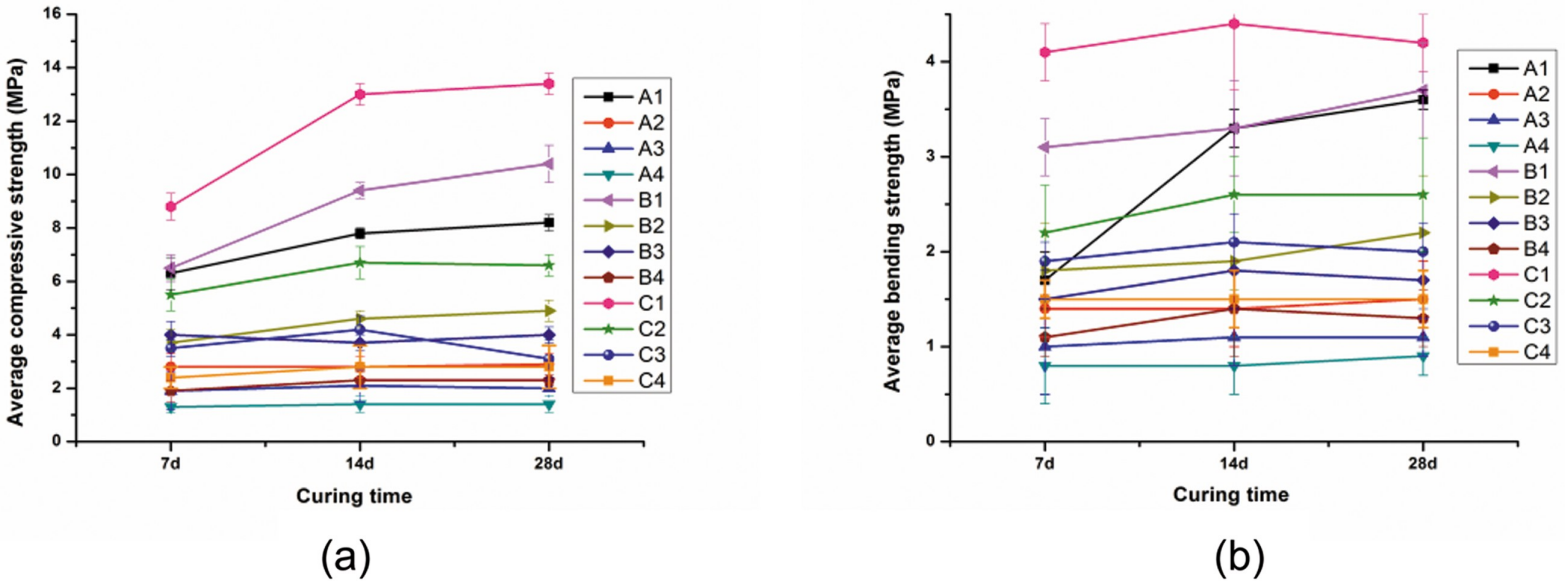
Relationship between curing time and average compressive strength (a), flexural strength (b).

In conclusion, average compressive strength and flexural strength of cement-based materials are greatly reduced with the increase of the content of rice husk, and the intensity of 7 ~ 28d is slow or negative when mass of cementitious materials was equal, as shown in Fig 9a. Under the same conditions of curing time, with the increase of the amount of cementitious material, corresponding to average compressive and flexural strength also increase (Fig 9b).

### 3.3. Influence of the content of rice husk fiber on thermal conductivity and apparent density of thermal insulation wallboards

The thermal conductivity of thermal insulation wallboards was tested and the results are shown in Table 2. In the absence of rice husk, thermal conductivity λ of A1, B1, and C1 is the largest under 28d of curing time. With the increase of rice hull content, thermal conductivity and apparent density of thermal insulation wallboards decrease correspondingly. Apparent density of thermal insulation wallboards is in range of 1680–1180 Kg/m^3^. When the content of rice hull is the largest, the value of thermal conductivity λ is minimum, indicating the better the insulation performance. The average compressive strength of the specimens should be greater than 4.0 MPa according to the actual engineering needs. Fig 8 shows B2 and C2 meet the engineering needs. According to the thermal conductivity, the coefficient of C10 is less than B2, as shown in Table 2. According to test data for C10, thermal insulation wallboards was prepared by mixing river sand (<3mm), cement, fly ash, cellulose ether, and naphthalene superplasticizer, and thermal conductivity of the wallboards 0.9600 W/m·°C at 28days. In actual engineering, materials with a thermal conductivity of less than 0.20 W/m·°C are usually called thermal insulation materials. Therefore, C2 can meet certain engineering requirements according to thermal conductivity and strength of thermal insulation wallboards [28–30].

**Table 2:** Thermal conductivity of test results at 28d.

Number	Cold plate temperature (°C)	Hot plate temperature (°C)	Apparent density (Kg/m^3^)	Specimen thickness (m)	Thermal conductivity (W/m·°C)
A1	15	35	1550	0.027	0.1852
A2	15	35	1290	0.027	0.1702
A3	15	35	1220	0.026	0.1433
A4	15	35	1190	0.024	0.1292
B1	15	35	1600	0.026	0.1932
B2	15	35	1450	0.026	0.1997
B3	15	35	1420	0.024	0.1741
B4	15	35	1180	0.025	0.1514
C1	15	35	1680	0.028	0.1958
C2	15	35	1490	0.029	0.1810
C3	15	35	1260	0.026	0.1671
C4	15	35	1230	0.028	0.1239

## 4. Conclusions

Thermal insulation wallboards can well be cast by the mixed slurry of cement, fly ash, cellulose ether, naphthalene superplasticizer, rice hull, and recycled concrete aggregates when slurry consistency was about 8CM. B2 and C2 specimens of compressive strength is bigger than 4MPa, and can be applied to actual engineering. The content of cementitious materials and rice hull has an important influence on the mechanical properties of the resulting thermal insulation wallboards, affecting their application. Average compressive strength, flexural strength, and thermal conductivity of the specimens decreased as the content of rice hull increased. Thermal conductivity of C2 was 0.1810 W/m·°C less than B2 (0.1997 W/m·°C) and blank group (0.9600 W/m·°C) when curing time was 28days. Therefore, C10 will be chosen and used in certain engineering.

## Supporting information

S1 Fig Recycled concrete aggregates. (TIF)

S1 Table Chemical composition of the ordinary Portland cement. (DOCX)

S2 Table Real content of water. (DOCX)
