# Supplementary figures and images for: Strength, microstructure, and thermal conductivity of the insulation wallboards prepared with rice husk fiber and recycled concrete aggregates
Source: PLoS One. 2018 Sep 19;13(9):e0203527. doi: 10.1371/journal.pone.0203527 (PMC6145573; doi:10.1371/journal.pone.0203527)

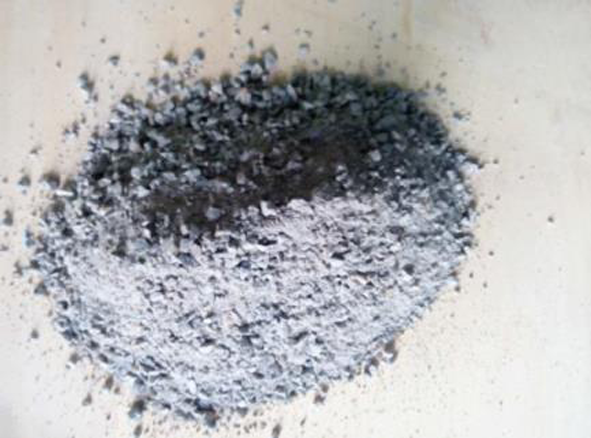

Supplement: S1 Fig — (TIF) [file pone.0203527.s001.tif]
