# Supplementary material for: Strength, microstructure, and thermal conductivity of the insulation wallboards prepared with rice husk fiber and recycled concrete aggregates
Source: PLoS One. 2018 Sep 19;13(9):e0203527. doi: 10.1371/journal.pone.0203527 (PMC6145573; doi:10.1371/journal.pone.0203527)
Supplement: S1 Table — (DOCX) [file pone.0203527.s002.docx]

**S1 Table. Chemical composition of the ordinary Portland cement.**

| SiO_2_ | Al_2_O_3_ | Fe_2_O_3_ | Cl^-^ | CaO | MgO | SO_3_ | R_2_O |
| --- | --- | --- | --- | --- | --- | --- | --- |
| 25.99 | 5.89 | 2.85 | 0.013 | 55.27 | 3.42 | 2.02 | 0.51 |
