# Supplementary material for: Strength, microstructure, and thermal conductivity of the insulation wallboards prepared with rice husk fiber and recycled concrete aggregates
Source: PLoS One. 2018 Sep 19;13(9):e0203527. doi: 10.1371/journal.pone.0203527 (PMC6145573; doi:10.1371/journal.pone.0203527)
Supplement: S2 Table — (DOCX) [file pone.0203527.s003.docx]

**S2 Table. Real content of water.**

| Number | Real content of water (g) |
| --- | --- |
| A1 | 4206.75 |
| A2 | 3501.25 |
| A3 | 3309.75 |
| A4 | 3223.75 |
|  |  |
| B1 | 4338.25 |
| B2 | 3925.25 |
| B3 | 3846.00 |
| B4 | 3186.50 |
|  |  |
| C1 | 4554.75 |
| C2 | 4039.25 |
| C3 | 3423.00 |
| C4 | 3345.00 |
